# Omitting patients with no follow-up leads to bias when using inverse-intensity weighted GEEs to handle irregular and informative assessment times

**DOI:** 10.1186/s12874-025-02721-z

**Published:** 2025-12-04

**Authors:** Xiawen Zhang, Anna Heath, Wei Xu, Eleanor Pullenayegum

**Affiliations:** 1 Dalla Lana School of Public Health, University of Toronto, 155 College Street, Toronto, Ontario M5T 3M7 Canada; 2 Child Health Evaluative Sciences, Hospital for Sick Children, 555 University Avenue, Toronto, Ontario M5G 1X8 Canada; 3 Princess Margaret Cancer Centre, University Health Network, 610 University Avenue, Toronto, Ontario M5G 2M9 Canada

**Keywords:** Longitudinal data, Informative observation, Inverse weighting, Generalized estimating equations

## Abstract

**Background:**

Longitudinal data can be used to study disease progression and are often collected at irregular intervals. When the assessment times are informative about the severity of the disease, regression analyses of the outcome trajectory over time based on Generalized Estimating Equations (GEEs) result in biased estimates of regression coefficients. Inverse-intensity weighted GEEs (IIW-GEEs) are a popular approach to account for informative assessment times and yield unbiased estimates of outcome model coefficients when the assessment times and outcomes are conditionally independent given previously observed data. However, a consequence of irregular assessment times is that some patients may have no follow-up assessments at all, and it is common practice to omit these patients from analyses when studying the outcome trajectory over time.

**Methods:**

We show mathematically that IIW-GEEs yield biased estimates of regression coefficients when patients with no follow-up assessments are excluded from analyses. We design a simulation study to evaluate how the bias varies with sample size, assessment frequency, follow-up time, and the informativeness of the assessment time process. Using the STAR*D trial of treatments for major depressive disorder, we examine the extent of bias in practice.

**Results:**

Our simulation results showed the bias incurred by omitting patients with no follow-up visits increased as visit frequency decreased and as the duration of follow-up decreased. In the STAR*D trial, omitting patients with no follow-up visits led to over-estimation of the rate of improvement in depressive symptoms.

**Conclusions:**

Studies should be designed to ensure patients with no follow-up are included in the data. This can be achieved by a) creating inception cohorts; b) when taking sub-samples of existing cohorts, ensuring that patients without follow-up assessments are included; c) dropping exclusion criteria based on availability of follow-up visits.

**Supplementary Information:**

The online version contains supplementary material available at 10.1186/s12874-025-02721-z.

## Background

Longitudinal data play a crucial role in studying prognosis and treatment effects over time. Outcomes are measured over time for each participant and studies typically aim to use these data to understand how outcomes evolve over time, or to assess whether specific treatments or patient characteristics lead to different outcome trajectories. In both observational and interventional studies, the times at which outcomes are assessed may be irregular, with the number of timing of assessments varying among subjects. This may occur, for example, if data is gathered through a chart review when all follow-up is part of usual care (see [1] for an example of a trial that used this design).

The timing of assessments is often related to study outcomes; for example, low weight gain in a neonate would trigger more frequent assessments. Dependence between outcomes and assessment frequency leads to standard methods of analysis such as Generalized Estimating Equations (GEEs) giving biased estimates of regression coefficients [2]. A range of methods have been proposed to handle irregular and informative assessment times, including inverse-intensity weighted generalized estimating equations (IIW-GEEs) [2] and semi-parametric joint models [3–6]; see [7] for a review. IIW-GEEs yield consistent inferences when the assessment time and outcome processes are conditionally independent given previously observed data, while semi-parametric joint models are appropriate when the assessment time and outcome processes are conditionally independent given random effects. In this paper we focus on IIW-GEEs as they are the most widely used [8], and unlike semi-parametric joint models can handle the common phenomenon of assessment times depending on a time-varying covariate.

A consequence of irregular assessment times is that there may be some patients with no follow-up at all. These patients are often excluded from analyses. For example, a review of prognosis studies in systemic lupus erythematosus found that 14% of studies used availability of the outcome as an inclusion criterion [9], and a review of longitudinal studies in older adults found that 75% of studies had exclusions due to lack of follow-up data [10].

Moreover, exclusion of patients with no follow-up may be unintentional and occurs through implicit inclusion criteria; this can happen when taking a subsample of an existing cohort or when creating a new cohort. For example, in studying the relationship between air quality and outoor play, Pullenayegum et al. [11, 12] used an inception cohort of children recruited through primary care clinics [13] and took a subsample of all measurements of outdoor play taken between the ages of 2 and 10 years. Consequently children who were enrolled in the original cohort but did not visit their doctor between the ages of 2 and the earlier of 10 years or the year data was cut were excluded from the dataset. A similar phenomenon can occur when creating a de novo cohort. For example, in studying the effect of statins on fasting glucose, Hadar et al. created their cohort by extracting fasting glucose measurements from an EHR database; thus only patients with a fasting glucose measurement were included in the dataset [14].

If the timing of assessments is unrelated to outcomes, these exclusions do not induce any selection bias. However, when the timing of assessments is related to outcomes, exclusion of patients with no follow-up raises concerns over bias because the assessment times are themselves informative about the outcome. While IIW-GEEs provide unbiased inferences when outcomes and assessment times are conditionally independent given previously observed data, this assumes that all patients are included in the analysis. The purpose of this paper is to examine whether exclusion of patients with no follow-up assessments causes bias in IIW-GEE estimates of regression coefficients. In the Theoretical results section we show theoretically that exclusion of patients with no follow-up will lead to bias in IIW-GEE estimates of regression coefficients. In the Simulation section we use simulation to explore factors influencing the extent of the bias, and in the Example: the STAR*D study section we use the Sequenced Treatment Alternatives to Relieve Depression (STAR*D) study [15] to illustrate the impact in practice. We conclude with a Discussion section by considering the implications for researchers dealing with data subject to irregular assessment times.

## Theoretical results

In this section we show that omitting subjects with no follow-up assessments results in biased estimates of the assessment process parameters and consequently biased IIW-GEE estimates of the regression coefficients for the mean outcome model.

### Notation

Suppose $Y_i(t)$ is the outcome for patient *i* at time *t*, with $t \in [0,\tau ]$, and that we wish to fit the marginal model

1 $$\begin{aligned} E(Y_i(t)\mid \textbf{X}_i(t)) = \textbf{X}_i(t)\varvec{\beta }_0 \end{aligned}$$

for a row vector of covariates $\textbf{X}_i(t)$ and corresponding regression coefficients $\varvec{\beta }_0$. We do not observe $Y_i(t)$ at every time point $t\in [0,\tau ]$, but rather only when the patient comes in for a assessment.

Let $N_i(t)$ denote the number of follow-up assessments for patient *i* by time *t*, and let $\Delta N_i(t) = \lim _{\delta \downarrow 0} (N_i(t) - N_i(t-\delta ))$; thus $\Delta N_i(t)$ is equal to 1 if patient *i* has a visit at time *t* and 0 otherwise, and we set $N_i(0)=0$. Suppose there is a set of observed covariates $\textbf{Z}_i(t)$ such that the outcome at time *t* is conditionally independent of whether a visit occurs at time *t* given the covariates at time *t*, i.e. $\Delta N_i(t) \perp \!\!\! \perp Y_i(t) \mid \textbf{Z}_i(t)$. The covariates $\textbf{Z}_i(t)$ may contain elements of $\textbf{X}_i(t)$ and past observed values of $Y_i(t)$, and may also contain auxiliary covariates not included in the outcome mean model. We assume that the assessment process intensity at time *t* conditional on $\textbf{Z}_i(t)$ follows a proportional hazards model, that is

2 $$\begin{aligned} \lambda (t;\textbf{Z}_i(t),\varvec{\gamma }_0) = \lim _{\delta \downarrow 0} \frac{E(N_i(t) - N_i(t-\delta )\mid \textbf{Z}_i(t))}{\delta } = \lambda _0(t){\textrm{exp}}(\textbf{Z}_i(t)\varvec{\gamma }_0), \end{aligned}$$

where the log hazard ratios $\varvec{\gamma }_0$ and the baseline hazard $\lambda _0$ are unknown.

### Inverse-intensity weighted GEEs

The usual GEE equations under working independence can be written as

3 $$\begin{aligned} \sum \limits _i\int _0^\tau \textbf{X}_i(t)^\prime (Y_i(t)-\textbf{X}_i(t)\varvec{\beta })dN_i(t)=0 \end{aligned}$$

These result in biased estimates of the outcome model regression coefficients $\varvec{\beta }_0$ if $Y_i(t)$ and $dN_i(t)$ are dependent given $\textbf{X}_i(t)$ because the expectation of the left-hand side is no longer zero [2]. However, consistent estimates of $\varvec{\beta }_0$ can be obtained by solving the inverse-intensity weighted GEEs [2, 16], i.e. by solving

4 $$\begin{aligned} U(\varvec{\beta };\varvec{\gamma }_0) = \sum \limits _i\int _0^\tau \textbf{X}_i(t)^\prime \frac{(Y_i(t)-\textbf{X}_i(t)\varvec{\beta })}{{\textrm{exp}}(\textbf{Z}_i(t)\varvec{\gamma }_0)}dN_i(t)=0 \end{aligned}$$

Lin et al. [2] show that the mean of $U(\varvec{\beta };\varvec{\gamma }_0)$ is zero, and that the solution $\hat{\varvec{\beta }}$ to $U(\varvec{\beta };\hat{\varvec{\gamma }})=0$ is consistent provided that $n^{1/2}(\hat{\varvec{\gamma }}-\varvec{\gamma })$ is *o*(1). Maximizing the Cox partial likelihood for $\varvec{\gamma }$ using the full dataset yields an $o(n^{-1/2})$-consistent estimate provided that the intensity model is correctly specified [17].

### Bias due to omission of subjects with no follow-up assessments

We now consider the impact of excluding patients with no follow-up assessments. Let $\varvec{\hat{\beta }}^{EV}$ and $\varvec{\hat{\gamma }}^{EV}$ be the estimates of $\varvec{\beta }$ and $\varvec{\gamma }$ on using everyone, and $\varvec{\hat{\beta }}^{FU}$ and $\varvec{\hat{\gamma }}^{FU}$ be the estimates of $\varvec{\beta }$ and $\varvec{\gamma }$ on omitting patients with no follow-up assessments. For any given $\varvec{\gamma }$, $U(\varvec{\beta };\varvec{\gamma })$ remains unchanged on omitting subjects with no follow-up assessments:

$$\begin{aligned} U(\varvec{\beta };\varvec{\gamma })= & \sum \limits _{i}\int _0^\tau \textbf{X}_i(t)^\prime (Y_i(t)-\textbf{X}_i(t)\varvec{\beta }){\textrm{exp}}(-\textbf{Z}_i(t)\varvec{\gamma })dN_i(t) \\= & \sum \limits _{i:N_i(\tau )>0}\int _0^\tau \textbf{X}_i(t)^\prime (Y_i(t)-\textbf{X}_i(t)\varvec{\beta }){\textrm{exp}}(-\textbf{Z}_i(t)\varvec{\gamma })dN_i(t) \end{aligned}$$

As excluding subjects with no follow-up assessments has no impact on the pseudo-score function $U(\varvec{\beta };\varvec{\gamma })$, any bias in $\varvec{\hat{\beta }}^{FU}$ induced by omitting patients with no follow-up assessments will occur due to bias in $\varvec{\hat{\gamma }}^{FU}$. In the special case where the assessment intensity covariates $\textbf{Z}_i$ and the baseline hazard $\lambda _0$ are time-independent, a Taylor expansion of $U(\varvec{\beta };\varvec{\hat{\gamma }}^{FU})$ about $\varvec{\gamma }_0$ yields, to first order,

$$\begin{aligned} & E(\varvec{\hat{\beta }}^{FU}-\varvec{\beta }_0) =-\left( \int _0^\tau E \left( \textbf{X}_i(t)^\prime \textbf{X}_i(t) \right) dt\right) ^{-1} \\ & E\left( \int _0^\tau E\left( \textbf{X}_i(t)^\prime (Y_i(t)-\textbf{X}_i(t)\varvec{\beta }_0)\mid \textbf{Z}_i\right) dt\textbf{Z}_iE(\hat{\gamma }^{FU}-\varvec{\gamma }_0\mid \textbf{Z}_i)\right) \end{aligned}$$

(see Appendix A for a general derivation). Thus if $\varvec{\hat{\gamma }}^{FU}$ is biased, $\varvec{\hat{\beta }}^{FU}$ will also be biased.

#### Bias in the inverse intensity weights

In Appendix A we give a general expression for the bias in $\varvec{\hat{\gamma }}^{FU}$. In the special case where the intensity covariates $\textbf{Z}_i$ and the baseline hazard $\lambda _0$ are time-invariant, this expression simplifies to

$$\begin{aligned} -\left( \left( \frac{s_1^*(\varvec{\gamma }_0)}{s_0^*(\varvec{\gamma }_0)}\right) ^2-\frac{s_2^*(\varvec{\gamma }_0)}{s_0^*(\varvec{\gamma }_0)}\right) ^{-1} \left( \frac{s_1^*(\varvec{\gamma }_0)}{s_0^*(\varvec{\gamma }_0)}- \frac{s_1(\varvec{\gamma }_0)}{s_0(\varvec{\gamma }_0)}\right) \end{aligned}$$

where

$$\begin{aligned} \begin{array}{ll} s_0(\varvec{\gamma }_0)=E({\textrm{exp}}(\varvec{\gamma }_0^{\prime } \textbf{Z}_i)) & s_0^*(\varvec{\gamma }_0)=E({\textrm{exp}}(\varvec{\gamma }_0^{\prime } \textbf{Z}_i)\mid N_i(\tau )>0) \\ \textbf{s}_1(\varvec{\gamma }_0)=E(\textbf{Z}_i{\textrm{exp}}(\varvec{\gamma }_0^{\prime } \textbf{Z}_i)) & \textbf{s}_1^*(\varvec{\gamma }_0)=E(\textbf{Z}_i{\textrm{exp}}(\varvec{\gamma }_0^{\prime } Z_j)\mid N_i(\tau )>0)\\ \textbf{s}_2(\varvec{\gamma }_0)=E(\textbf{Z}_i\textbf{Z}_i^\prime {\textrm{exp}}(\varvec{\gamma }_0^{\prime } \textbf{Z}_i)) & \textbf{s}_2^*(\varvec{\gamma }_0)=E(\textbf{Z}_i\textbf{Z}_i^\prime {\textrm{exp}}(\varvec{\gamma }_0^{\prime } \textbf{Z}_i)\mid N_i(\tau )>0)\\ \end{array} \end{aligned}$$

Since both $\textbf{Z}_i$ and $\lambda _0$ are time-invariant, $N_j(\tau )\mid \textbf{Z}_i\sim \text {Poisson}( \lambda _0{\textrm{exp}}(\varvec{\gamma }_0^\prime \textbf{Z}_i)\tau )$. It follows that if $\varvec{\gamma }_0$ is positive, when we omit patients with no follow-up assessments we will tend to see larger values of the covariates $\textbf{Z}_i$, so that $s_0^*>s_0$ and $\textbf{s}_1^*>\textbf{s}_1$. In fact it is possible to evaluate the conditional expectations in the expressions for $s_0^*$ and $s_1^*$ given the distribution of $\textbf{Z}$ analytically. Here we consider $Z_i\sim$ Bernoulli(0.5), and provide results for $Z_i\sim$ Normal(0,1) and $Z_i\sim$ Gamma(1,1) in Appendix A.

**Fig. 1:**
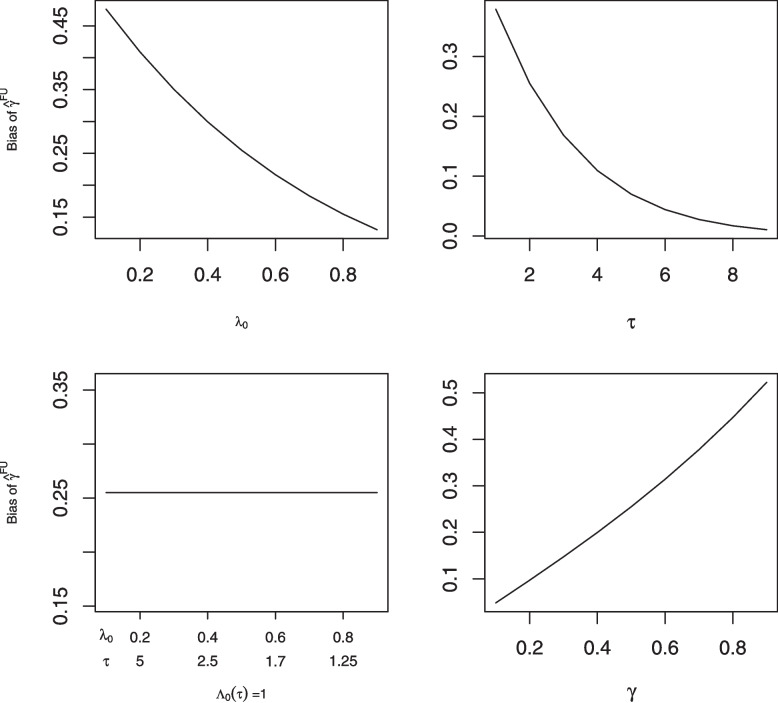
Bias in the regression coefficient $\hat{\gamma }$ in the assessment intensity model for a single time-invariant covariate $Z_j \sim$ Bernoulli(0.5) as: the time-invariant assessment intensity $\lambda _0$ is varied (top left); the total follow-up time $\tau$ is varied (top right); $\lambda _0$and $\tau$ are varied while holding $\Lambda _0(\tau )(=\lambda _0 \times \tau )$ fixed (bottom left); $\gamma$ is varied while decreasing $\lambda _0$ to hold the expected number of assessments constant (bottom right)

As can be seen from Fig. 1, the magnitude of the bias of $\hat{\gamma }^{FU}$ decreases as either the baseline hazard $\lambda _0$ or the follow-up time $\tau$ increases. If $\lambda _0$ and $\tau$ are changed while keeping $\Lambda _0(\tau )=\lambda _0\tau$ constant, the bias of $\hat{\gamma }^{FU}$ remains unchanged. This makes sense, since the probability of no follow-up assessments is ${\textrm{exp}}(-\lambda _0\tau {\textrm{exp}}(\gamma _0^\prime Z_i))$, which decreases as either $\lambda _0$ or $\tau$ increases. Moreover, $\lambda _0$ and $\tau$ affect the probability of a subject having no follow-up assessments only through their product.

Increasing $\varvec{\gamma }_0$ while decreasing $\lambda _0$ to keep the mean number of assessments constant leads to increased bias of $\hat{\gamma }^{FU}$ (Fig. 1, bottom right panel). Intuitively this makes sense because when $\varvec{\gamma }_0$ is zero the assessment process is independent of $\textbf{Z}_i$ and we would not expect omission of subjects with no follow-up assessments to lead to bias.

Similar results hold for $Z_i\sim$ Normal(0,1) and $Z_i\sim$ Gamma(1,1) (see Appendix A).

## Simulation

### Simulation set-Up

While closed-form expressions for the bias in $\varvec{\hat{\beta }}^{FU}$ are available when the intensity model covariates $\varvec{Z}$ are time-invariant, this is not the case for time-varying $\varvec{Z}$. We used a simulation study that aimed to examine the bias when omitting subjects with no follow-up assessments when $\varvec{Z}$ is time-varying. Specific hypotheses are outlined in Table 1.

**Table 1:** Simulation parameters, hypotheses and results. Unless otherwise specified, $\lambda _0=0.5$, $\varvec{\gamma }_0=0.5$, $n=500$, $\tau =2$. We consider two data-generating mechanisms, one with $E(Y(t)) = \mu _{01}(t) = 3.3+\frac{4}{(1+t)^2} + 10.5\frac{log(1+t)}{(1+t)^2}$ and the other with $E(Y(t))=\mu _{02}=3.3$

Hypothesis			Results	
Change	Bias of	Parameter	$E(Y(t))=$	
	$\hat{\gamma }^{FU}$&$\hat{AUC}^{FU}$	values	$\mu _{01}(t)$	$\mu _{02}$
Increasing$\lambda _0$	$\downarrow$	$\lambda _0=$0.1, 0.3, 0.5, 0.7, 0.9	$\checkmark$	$\checkmark$
Increasing$\tau$	$\downarrow$	$\tau =$1, 1.5, 2.0, 2.5, 3	$\checkmark$	$\checkmark$
Increasing$\varvec{\gamma }_0$while decreasing$\lambda _0$to hold$P(N_i(\tau )=0)$fixed	$\uparrow$	$\gamma =$0, 0.2, 0.4, 0.6, 0.8	✗	$\checkmark$
Increasing *n* when *n* is large enough that the bias in$\hat{\gamma }^{EV}$is small	no effect	$n=$100, 200, 300, 400, 500	$\checkmark$	$\checkmark$
Increasing *n* when *n* is small enough that the bias of$\hat{\gamma }^{EV}$is non-negligible	$\downarrow$	$n=$10, 20, 30, 40, 50	$\checkmark$	✗

We parameterized our simulation study using a study of intravenous immunoglobulin for the treatment of juvenile dermatomyositis (JDM) [18]. The primary outcome of the study was a modified disease activity score [19], with higher scores indicating worse disease activity; scores range from 0–12 and although slightly non-Normal we assumed Normality in our simulation for simplicity. Taking $T_{ij}$ to be the follow-up time of subject *i* at the $j^{th}$ assessment, we followed [20], in setting the the assessment intensity to be

$$\begin{aligned} \lambda _i(t)=\lambda _0{\textrm{exp}}(\gamma \log (1+Y_i(T_{iN_i(t^-)}))), \end{aligned}$$

i.e., the assessment intensity depends on the value of the outcome at the last visit.

We considered two data-generating mechanisms, one in which the mean of the outcome was time-varying, and one in which the mean of the outcome was time-invariant. Specifically, setting $X_1(t) = \frac{1}{(1+t)^2}$, $X_2(t) = \frac{\log (1+t)}{(1+t)^2}$ and letting $E(Y_i(t))=\mu (t)$, we took:

$$\begin{aligned} \mu (t)= & \begin{array}{ll} \mu _{01}(t) = 3.3 + 4X_1(t) + 10.5 X_2(t) & \text { (data generating mechanism 1)}\\ \mu _{02} = 3.3 & \text { (data generating mechanism 2)} \end{array} \\ Y_i(t)= & \mu (t) + u_i + v_it +\epsilon _i(t)\quad \text{ with}\\ \left( \begin{array}{c} u_i \\ v_i \end{array}\right)\sim & \text {Multivariate Normal }\left( 0, \left( \begin{array}{cc} 1.6^2 & -0.7\times 1.6\times 1.2\\ -0.7\times 1.6\times 1.2 & 1.2^2 \end{array}\right) \right) \end{aligned}$$

where the residual $\epsilon _i(t)$ followed an exponential correlation structure (standard deviation $=1.5$, range $=0.5$, nugget $=0.4$). Since the assessment intensity depends on the last observed outcome, we began by simulating outcomes at baseline and thereafter alternated between simulating visit times given the last observed outcome and outcome given visit time.

Our estimand was the estimated AUC, i.e. the area under the curve when mean disease activity score is plotted against time (AUC = $\int _0^\tau \mu (t;\varvec{\beta })dt$); the AUC indicates the average burden of disease over the time interval $[0,\tau ]$. For $E(Y(t)) = \mu _{01}(t)$, the true value of the AUC is $3.3\tau -4.0\frac{1}{1+\tau }+10.5\frac{\ln (1+\tau )+1}{1+\tau }-6.5$; for $E(Y(t))=\mu _{02} = 3.3$, it is $3.3\tau$. Our main performance measure was bias of the estimated AUC on omitting subjects with no follow-up assessments ($\hat{AUC}^{FU}$). To aid interpretation we also examined the bias of $\hat{\gamma }^{FU}$, and for the purposes of comparison considered the bias of the estimated AUC on including everyone $\hat{AUC}^{EV}$, and the bias of $\hat{\gamma }^{EV}$.

Each simulated dataset was analyzed using IIW-GEEs, regressing outcomes onto $X_1(t)$ and $X_2(t)$, first including everyone then excluding patients with no follow-up assessments. We used 5000 iterations for each set of parameter values and each data generating mechanism. All data generation and modelling was performed using R 4.4.0 [21].

### Simulation results

**Fig. 2:**
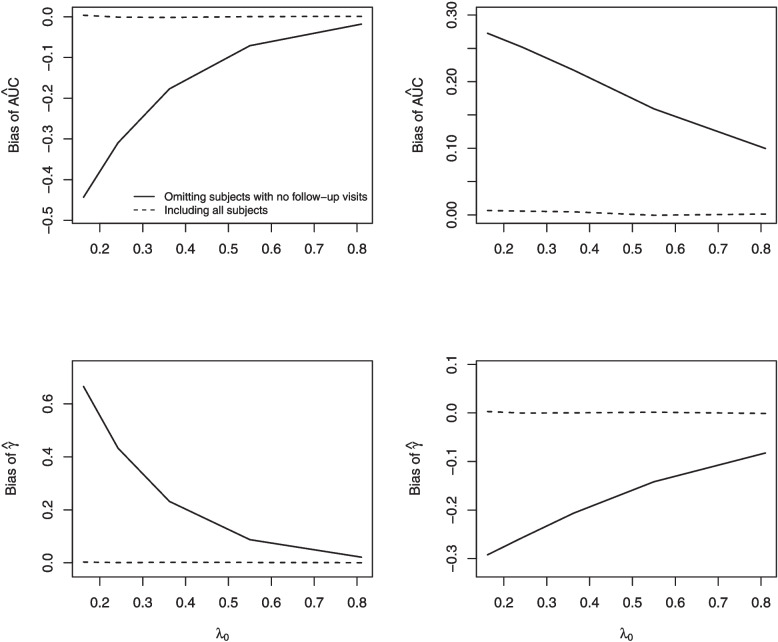
The relationship between bias ($\hat{\text {AUC}}$ and $\hat{\gamma }$) and $\lambda _0$. Figures in the left column are for $E(Y(t)) = 3.3 + \frac{4.0}{(1+t)^2} +10.5\frac{\log (1+t)}{(1+t)^2}$ and figures in the right column are for $E(Y(t))=3.3$

**Fig. 3:**
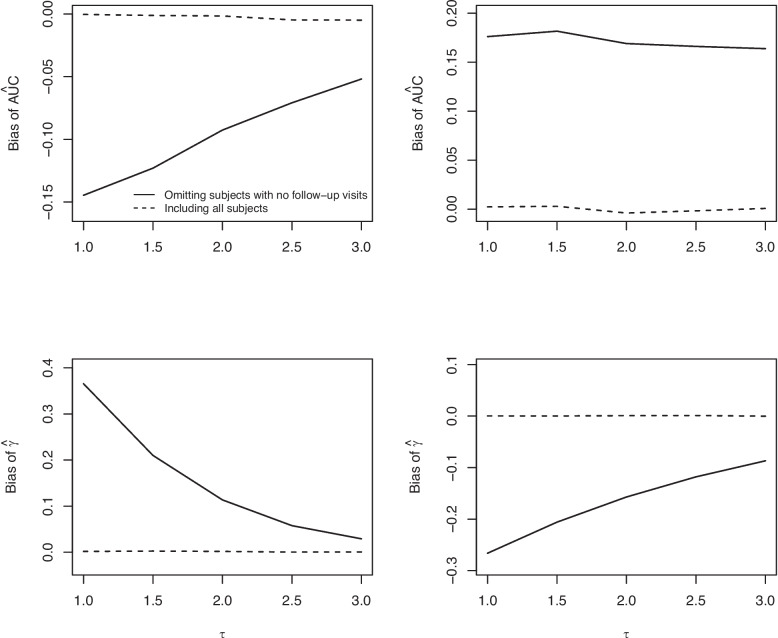
The relationship between bias ($\hat{\text {AUC}}$ and $\hat{\gamma }$) and $\tau$. Figures in the left column are for $E(Y(t)) = 3.3 + \frac{4.0}{(1+t)^2} +10.5\frac{\log (1+t)}{(1+t)^2}$ and figures in the right column are for $E(Y(t))=3.3$

Increasing either $\lambda _0$ or $\tau$ decreased the bias of $\hat{\text {AUC}}^{FU}$ and $\hat{\gamma }^{FU}$ (see Figs. 2, 3). When $E(Y(t))=\mu _{01}(t)$ the bias in the estimated AUC was negative, whereas when $E(Y(t))=3.3$ it was positive.

**Fig. 4:**
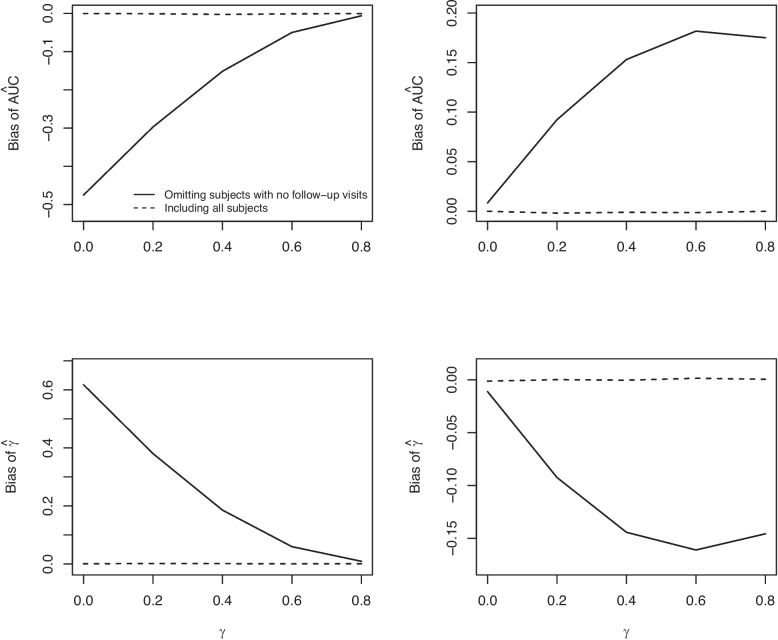
The relationship between bias ($\hat{\text {AUC}}$ and $\hat{\gamma }$) and $\gamma$. Figures in the left column are for $E(Y(t)) = 3.3 + \frac{4.0}{(1+t)^2} +10.5\frac{\log (1+t)}{(1+t)^2}$ and figures in the right column are for $E(Y(t))=3.3$

When $E(Y(t))=\mu _{01}(t)$, the magnitude of the bias of $\hat{\text {AUC}}^{FU}$ and $\hat{\gamma }^{FU}$ decreased with increasing $\varvec{\gamma }_0$ and was non-zero when $\varvec{\gamma }_0=0$ (Fig. 4). When $E(Y(t))=3.3$, the bias increased with increasing $\varvec{\gamma }_0$, and was approximately zero when $\varvec{\gamma }_0=0$.

Once sample size was sufficient that the bias of $\hat{\gamma }^{EV}$ was small, further increasing the sample size did not affect the bias in either $\hat{\gamma }^{FU}$ or $\hat{\text {AUC}}^{FU}$ on excluding subjects with no follow-up (see Appendix Fig. 11).

**Fig. 5:**
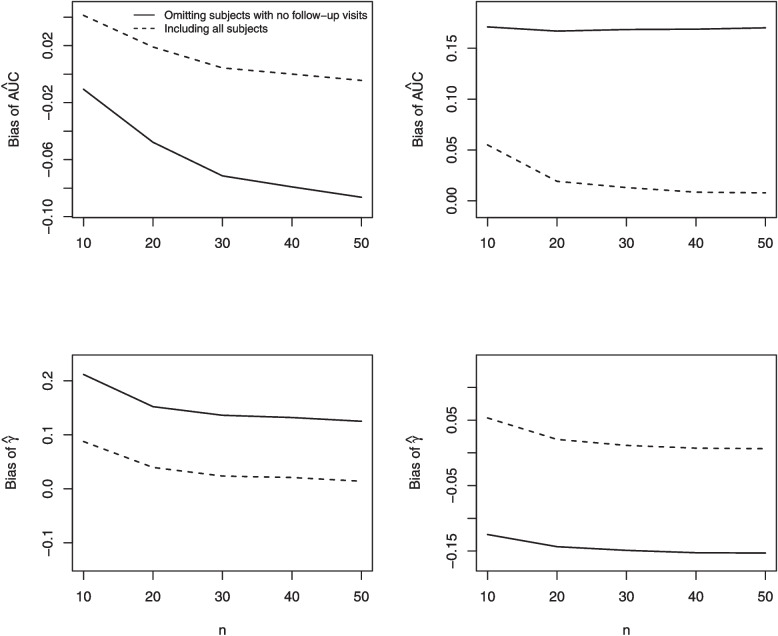
The relationship between bias ($\hat{\text {AUC}}$ and $\hat{\gamma }$) and small *n*, figures in the left panel are for data-generating mechanism 1 and figures in the right panel are for data-generating mechanism 2

When sample sizes were small enough that $\hat{\gamma }^{EV}$ was biased, increases in sample size reduced the bias in $\hat{\gamma }^{FU}$ and $\hat{\text {AUC}}^{FU}$ when $E(Y(t))=\mu _{01}(t)$, but had little effect on the bias in $\hat{\text {AUC}}^{FU}$ when $E(Y(t))=3.3$ (Fig. 5). Looking at the bias in $\hat{\gamma }^{EV}$ we see that for $E(Y(t))=\mu _{01}(t)$ the bias due to the sample size being small was positive but reduced with increasing *n* so that at $n=50$ it was close to zero. Looking at $\hat{\gamma }^{FU}$ at $n=50$ we see that the bias in $\hat{\gamma }^{FU}$ is positive, indicating that the bias due to omitting subjects with no follow-up assessments is also positive. Making these same comparisons for $E(Y(t))=3.3$, the small sample bias is still positive, however the bias due to omitting subjects with no follow-up assessments is negative.

Tabulated simulation results, including empirical standard errors, are available in Appendix B. Empirical standard errors were very similar on including everyone vs. omitting people with no follow-up assessments; in cases where they differed, the standard errors on including everyone were smaller than those on omitting people with no follow-up assessments.

## Example: the STAR*D study

We showed the impact of omitting patients with no follow-up assessments through the Sequenced Treatment Alternatives to Relieve Depression (STAR*D) study [22]. STAR*D was a large randomized clinical trial to evaluate the effectiveness of the different treatments of major depressive disorder [15, 23]. For the purposes of illustration, we focussed on the first 16 weeks of level 1 of the study, in which everyone was treated with Citalopram. The protocol specified assessment times were 2, 4, 6, 9 and 12 weeks after enrolment, however there were both missed assessments and additional assessments due to patient need (see Fig. 12 in Appendix C). The Quick Inventory of Depressive Symptomatology (clinician-rated) (QIDS) [22] was recorded at every clinical assessment, where higher QIDS scores represent more severe depression. This analysis focussed on the trajectory of mean QIDS score over time.

We began by modeling the assessment intensity using an Andersen-Gill model in order to obtain inverse-intensity weights. The assessment intensity model used baseline characterstics as well as the last observed QIDS score; variables were retained in the model regardless of statistical significance. The log intensity ratio for last observed QIDS score was time-varying (see Appendix C Fig. 13), which was accommodated in the intensity model using the tt transform in the coxph function in R. Multiple imputation (MI) was used to handle missing baseline data.

The mean QIDS score declined over time in a non-linear manner, so we used the best-fitting fractional polynomial (with up to two time transforms) [24], as indicated by the adjusted R-squared. We modelled the outcome trajectory as a function of time alone in order to estimate the total burden of depressive symptoms over the 16 weeks (i.e., the area under the curve when mean QIDS score was plotted against time). We also examined which baseline variables affected the outcome trajectory by adding both the baseline covariates and their interactions with the fractional polynomial to the model; for this model we studied the change in QIDS score from baseline, as this is more meaningful when studying patient-specific effects. All interactions were added simulatenously and retained regardless of statistical significance. Parameters in these regression models were estimated through IIW-GEEs using the geeglm() function [25].

We conducted the analysis (a) including all patients and (b) excluding those without follow-up QIDS scores.

### Results

Our analysis included 4041 patients with a mean of 3.38 assessments (IQR 2 to 5, min 0, max 9). There were 481 (12%) patients with no follow-up assessments. Demographic characteristics are provided in Appendix C Table 6.

**Table 2:**
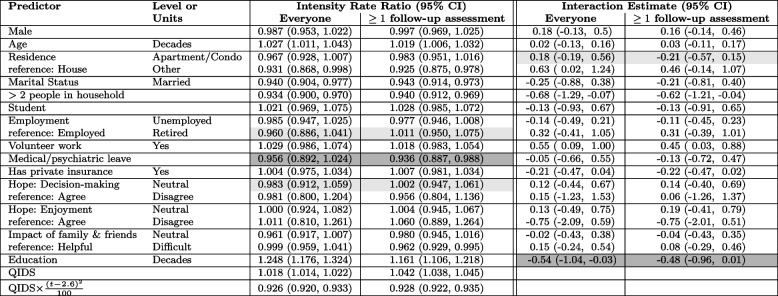
Intensity rate ratios for the fitted assessment intensity models and interaction effects for the QIDS model, fitted through inverse-intensity weighting. Light grey shading indicates covariates where the direction of association changed on restricting to those with at least one assessment, and dark grey shading indicates covariates where significance at the 5% level changed on restricting to those with at least one assessment. CI: Confidence Interval; QIDS: Quick Inventory of Depressive Symptomology

The assessment intensity model is given in Table 2. Regardless of whether patients with no follow-up were retained in the model, males visited less frequently than females, whereas those who were married visited more frequently. On including everyone, those who were retired visited less frequently than those who were employed, as did those who did not express hope for improvement in their decision-making, however these associations were reversed on excluding patients with no follow-up assessments. Those who were on medical or psychiatric leave visited less frequently regardless of whether those with no follow-up assessments were excluded; the association was not statistically significant on including everyone but became statistically significant on excluding those with no follow-up assessments.

**Fig. 6:**
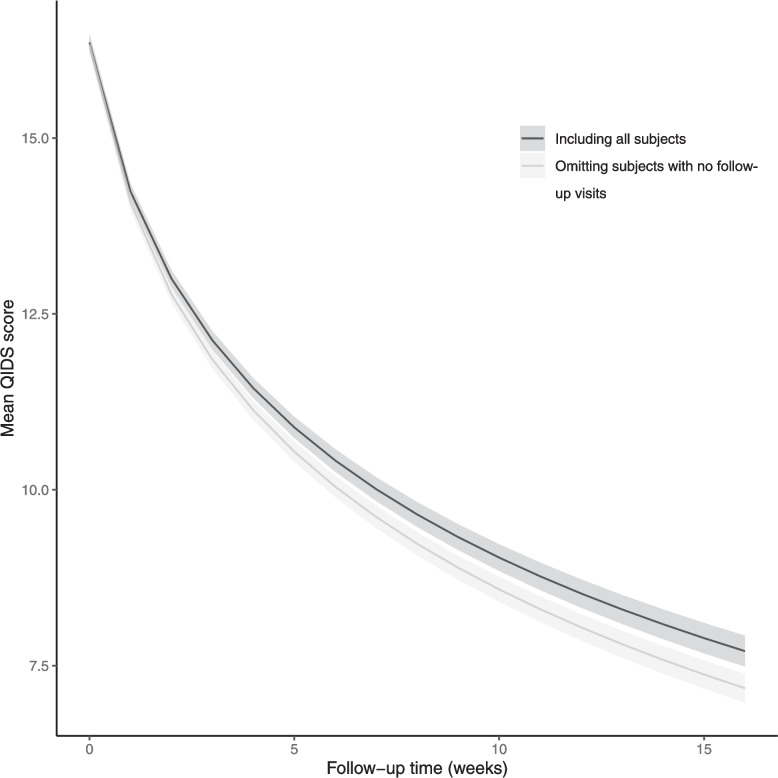
Mean QIDS score trajectory over time including all subjects and omitting subjects with no follow-up assessments. The shaded regions correspond to 95% confidence intervals for the mean QIDS score trajectory. The fitted mean model on including everyone is $\text {mean}(\text {QIDS}(t))=16.4 -3.1\log (1+t)$ with standard errors 0.061 and 0.042 for the intercept and $\log (1+t)$ respectively. On omitting people with no follow-up assessments the fitted model becomes $\text {mean}(\text {QIDS}(t))=16.3 -3.2\log (1+t)$ with standard errors 0.064 and 0.040 respectively

**Fig. 12:**
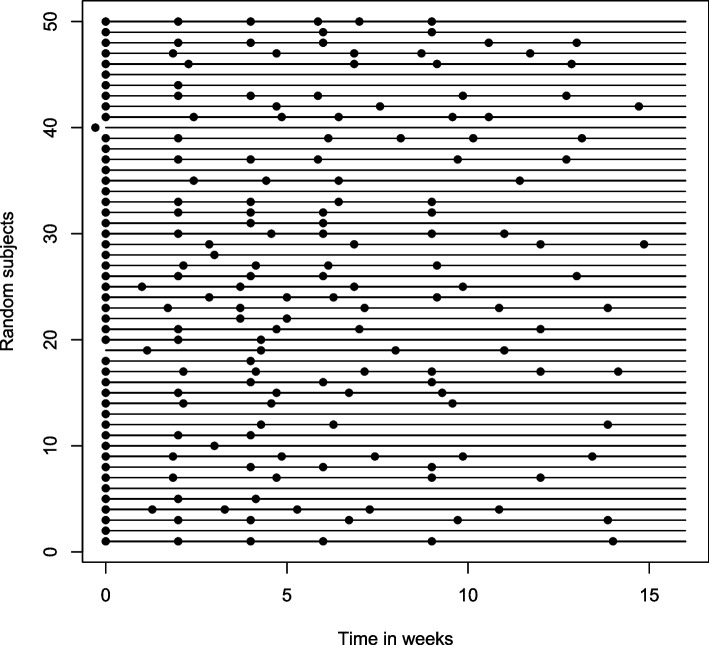
Follow-up times of random subset of 50 subjects in level 1

**Fig. 13:**
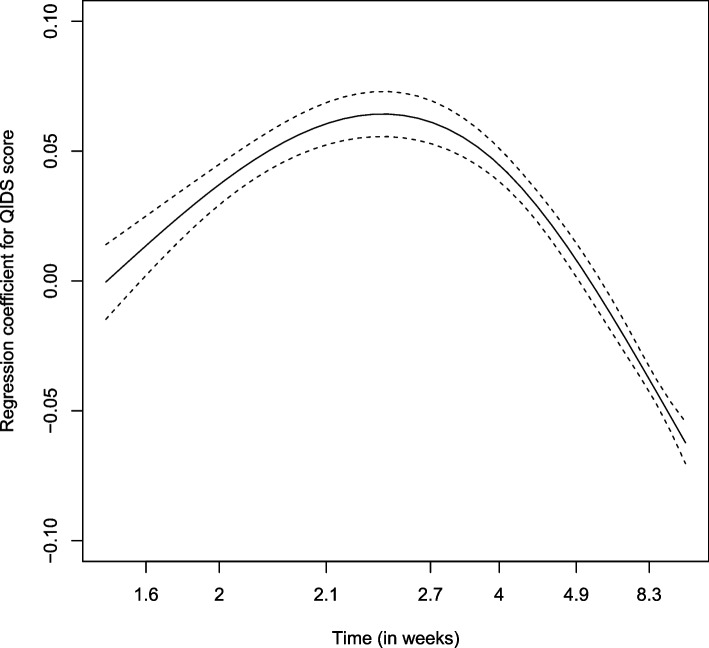
Log intensity ratio for QIDS score over time in Cox model

The estimated trajectories of QIDS scores are given in Fig. 6. While both trajectories decrease over time, omitting patients with no follow-up assessments leads to steeper estimates of the rate of decline. The AUC when mean QIDS score is plotted against time is 162 (standard error (SE) 1.32) on including everyone and 157 (SE 1.25) on excluding those with no follow-up assessments.

Interactions between baseline variables and $\log (1+\text {time})$ are given in Table 2 (see Appendix C Table 8 for main effects from this model). Patients living in households with more than two people and patients engaged in volunteer work experienced slower declines in QIDS scores (as evidenced by positive interaction effects); excluding patients with no follow-up assessments led to smaller estimates of the interactions (0.52 in excluding patients with no follow-up assessments vs. 0.57 on including everyone for $>2$ person households and 0.45 vs. 0.55 for volunteer work). More years of education were associated with more rapid declines in QIDS scores; the estimated interactions were smaller in magnitude on omitting patients with no follow-up assessments (−0.48 vs. −0.54), and moreover, the interaction effect lost statistical significance at the 5% level on excluding patients with no follow-up assessments.

## Discussion

While it is common practice to omit patients with no follow-up data in longitudinal studies [9, 10], we have shown, both theoretically and empirically, that omitting patients with no follow-up assessments from analyses using IIW-GEEs may lead to bias.

Our simulation study demonstrated that the magnitude of the bias can be difficult to predict when there are time-varying predictors of assessment intensity; our pre-specified hypotheses around the relationship between bias and $\varvec{\gamma }_0$ and *n* (for small *n*) were both false for one of the data generating mechanisms.

Specifically, when the mean of the outcome was time varying, we found that even when the assessment process was completely at random (i.e. $\varvec{\gamma }_0=0$), fitting an IIW-GEE excluding patients with no follow-up assessments induced bias. We believe that this is because when $E(Y_i(t))$ decreases over time, the expectation of the last observed $Y_i(t)$ decreases when we condition on $N_j(\tau )>0$, inducing a relationship between $N_i(t)$ and the last observed outcome. It is for this reason that we see bias in $\hat{\gamma }^{FU}$ when *E*(*Y*(*t*)) is time varying, but not when *E*(*Y*(*t*)) is constant.

For a time-constant *E*(*Y*(*t*)), when the sample size was small enough that $\hat{\gamma }^{EV}$ was biased, the bias in $\hat{\gamma }^{FU}$ increased rather than decreased as sample size increased. This may be due to the small sample size bias in $\hat{\gamma }$ acting in the opposite direction to bias due to omitting people with no follow-up assessments, so that they partially cancel one another out; when the sample size is increased the bias due to small sample size decreases so that the total bias in fact increases. Figure 5 supports this: the small sample bias is positive while the bias due to omitting patients with no follow-up assessments is negative. We do not see this cancelling out when $E(Y(t))=\mu _{01}(t)$ because both biases are positive.

In addition to the magnitude of the bias being unpredictable, as exemplified in the STAR*D study the extent of the bias can be non-trivial: omitting patients with no follow-up assessments leads to differing conclusions about predictors of assessment intensity, and also the trajectory of depressive symptoms over time.

The issue of exclusion of patients with no follow-up data also occurs in the context of regular observation (i.e., repeated measures data); this would happen when some patients miss all their pre-specified follow-up assessments. This has been widely studied in the missing data literature (see e.g. [26]). In a repeated measures study it is likely that at least some baseline data is available on everyone, making either multiple imputation [27, 28] or inverse-probability weighting [29, 30] (or a combination [31]) suitable approaches provided missingness is at random. In the presence of missingness not at random, approaches to global sensitivity analysis have been developed [32].

Despite its similarities with missing data, irregular observation is a much less recognised problem. Analytic solutions have been proposed, but as we have illustrated here in the context of IIW-GEEs, exclusion of patients with no follow-up data from the dataset prevent these methods from being applied appropriately. While we have studied only IIW-GEEs, we note that semi-parametric joint models rely on estimating equations that are zero mean [3–6], and that these equations lose their zero-mean property when analysis is restricted to individuals with follow-up assessments, and are thus also subject to bias. The same principle holds for fully parametric joint models [33–35], where omission of individuals with no follow-up assessments will lead to biased estimation of the intensity model. Furthermore, while we have focussed on cases where the stochastic nature of the visit process leads to some individuals having no follow-up assessments, there are also cases where individuals drop out of the study or data collection process, possibly informatively so. This issue has received limited attention and approaches to handling informative dropout in the presence of irregular observation are needed [36].

**Table 3:** Recommendations, by reason for exclusion of people with no follow-up data

Study design	Reason for exclusion	Design solution
Cohort study	Explicit exclusion criterion	Drop exclusion criteria based on number of follow-up assessments
Sub-study of existing cohort	Implicit inclusion criterion: patients must have a assessment in a specified time window in order to be included in the data cut	Include everyone with the desired baseline features in the data cut, regardless of whether there are follow-up assessments or not
De novo prevalent cohort	Implicit inclusion criterion: patients must have outcome assessed at an assessment in order to be recruited into the study	Prioritize population-based cohort studies of healthy individuals and inception cohorts among those with disease

It is therefore important that patients with no follow-up assessments be included in analyses. This is straightforward to do provided that they were in the dataset when it was originally cut. Specifically, we maintain them in the dataset when estimating the intensity model, noting that between the date they entered the study and the date of censoring (administrative or otherwise), there was no follow-up assessment. Detailed guidance on how to do this, with a sample dataset, code, and an R Markdown file is included in the Supplementary material. However, if patients are included in the dataset only if they have an observation in some pre-defined window, the problem is much harder to rectify. For example, in the study of outdoor play and air quality described in the introduction, we would need a new cut of the data including everyone enrolled in the study who was at least 2 years old at the date the data was cut; this would cost thousands of dollars. In other cases, where there is no parent cohort but we are instead creating a de novo prevalent cohort, it may be impossible to obtain data on patients who have no assessments. For example, the PROactive cohort [37] studies children with chronic disease at high risk of fatigue, decreased participation in daily life and psychosocial problems. Recruitment occurs at outpatient visits and the primary outcomes are assessed through patient-reported outcome measures longitudinally when patients attend their outpatient visits. Thus patients are recruited into the study only if they have an outpatient visit. In such prevalent cohort studies, there will typically be no way of knowing how many patients had no assessments, or how they differ in terms of demographic or health profiles from those patients included in the dataset.

One solution to this problem is to build large population-based cohorts (i.e., recruited when healthy) and inception cohorts (i.e., created at disease onset) with broad objectives and rich information collected from each participant; some of the research questions to be addressed in these cohorts will be posed *a priori*, however a particular strength of this design is that these cohorts can also be used to address questions that arise later. For example, the TARGetKids! study recuited children aged 0–5 years and is following them longitudinally until age 18, with the aim of identifying early life exposures predictive of later cardiometabolic risk [13]. The richness of the data collected at longitudinal follow-ups has allowed analyses beyond the originally posed questions, for example the association between early childhood nutritional risk and school readiness [38]. Similarly, the Canadian Longitudinal Study of Aging began in 2009 and recruited over 50,000 individuals aged 45–85 who are being followed longitudinally, with the overarching aim of identifying reasons why some people age in a healthy fashion whereas others do not [39]. The data are currently being used to study the impact of the COVID-19 pandemic on obesity and diabetes among older adults [40], a question that could not have been posed when the study was first initiated. Such population-based inception cohorts avoid the exclusion of patients with no outcome assessments. Table 3 provides a summary of our recommendations around designing studies to ensure that patients with no follow-up are included in the data.

In conclusion, excluding patients with no follow-up assessments results in biased estimates of regression coefficients. Consequently, researchers should ask themselves whether their study design requires patients to have the outcome assessed in a specific window in order to be included; this criterion may be present even if not explicitly stated. Wherever possible, patients should be included regardless of whether or not they had outcome assessments. If this is not possible, estimates of outcome trajectories should be interpreted with caution given the potential for bias.

## Supplementary Information

Additional file 1. Simulation code.

## Figures and Tables

**Fig. 7:**
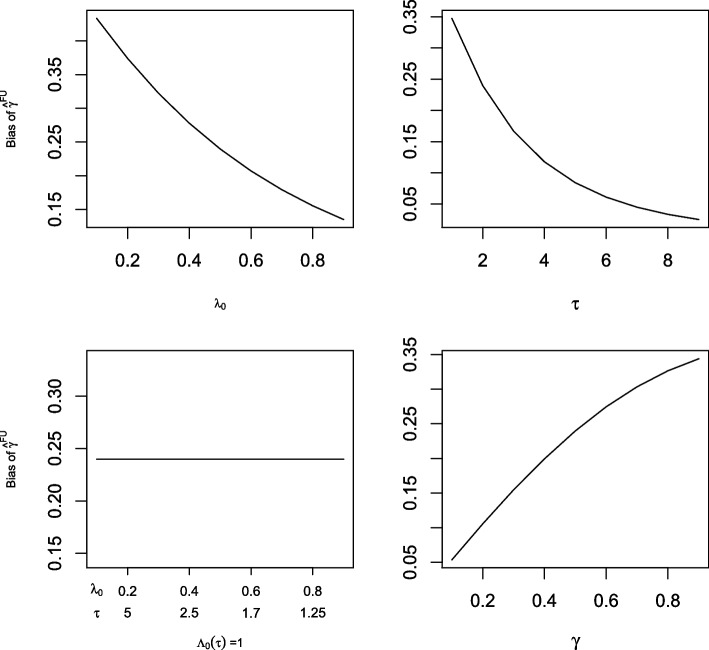
Bias in the regression coefficient $\hat{\gamma }$ in the assessment intensity model for a single time-invariant covariate $Z_j \sim$ Normal(0,1) as (**a**) the time-invariant assessment intensity $\lambda _0$ is varied (top left), **b** the total follow-up time $\tau$ is varied (top right), **c**$\lambda _0$ and $\tau$ are varied while holding $\Lambda _0(\tau )(=\lambda _0 \times \tau )$ fixed (bottom left), and (**d**) $\gamma$ is varied while decreasing $\lambda _0$ to hold the expected number of assessments constant

**Fig. 8:**
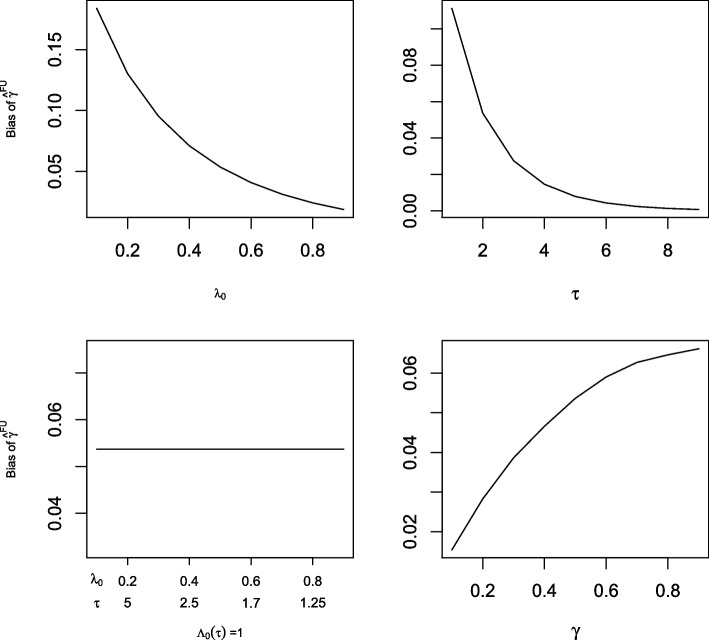
Bias in the regression coefficient $\hat{\gamma }$ in the assessment intensity model for a single time-invariant covariate $Z_j \sim$ Gamma(1,1) as (**a**) the time-invariant assessment intensity $\lambda _0$ is varied (top left), **b** the total follow-up time $\tau$ is varied (top right), **c**$\lambda _0$ and $\tau$ are varied while holding $\Lambda _0(\tau )(=\lambda _0 \times \tau )$ fixed (bottom left), and (**d**) $\gamma$ is varied while decreasing $\lambda _0$ to hold the expected number of assessments constant

**Fig. 9:**
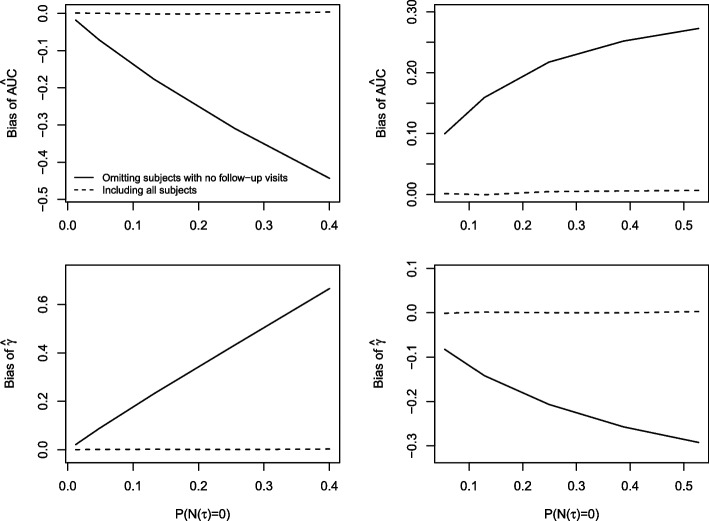
The relationship between bias ($\hat{\text {AUC}}$ and $\hat{\gamma }$) and P$(N_i(\tau )=0)$ by changing $\lambda _0$, figures in the left panel are for data-generating mechanism 1 and figures in the right panel are for data-generating mechanism 2

**Fig. 10:**
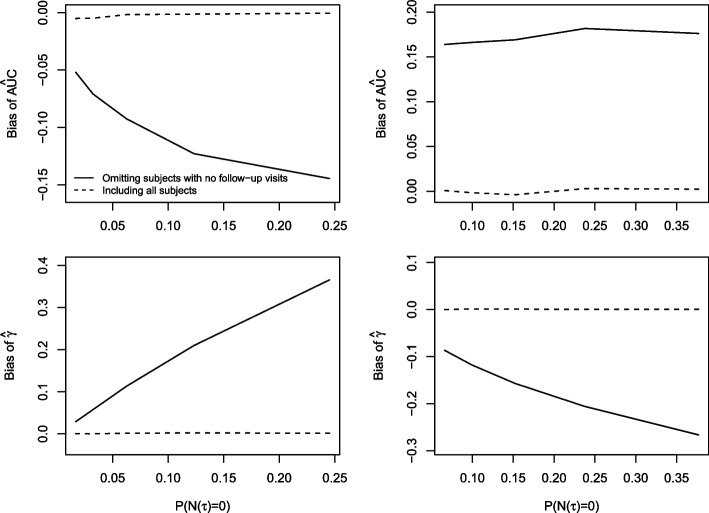
The relationship between bias ($\hat{\text {AUC}}$ and $\hat{\gamma }$) and P$(N_i(\tau )=0)$ by changing $\tau$, figures in the left panel are for data-generating mechanism 1 and figures in the right panel are for data-generating mechanism 2

**Fig. 11:**
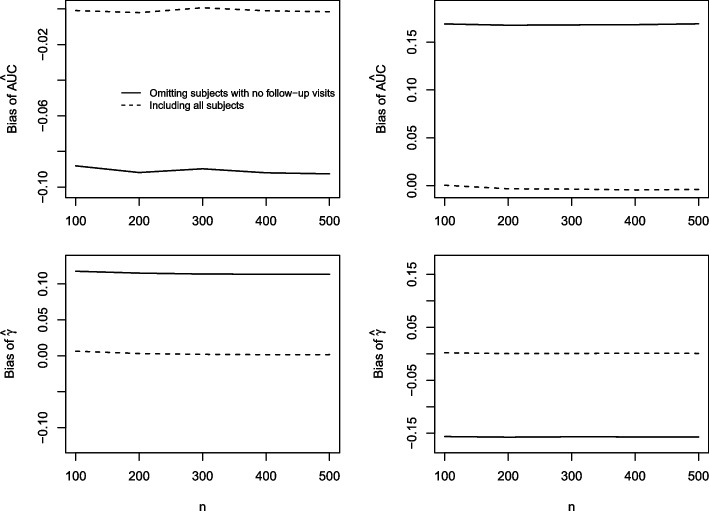
The relationship between bias ($\hat{\text {AUC}}$ and $\hat{\gamma }$) and large *n*, figures in the left panel are for data-generating mechanism 1 and figures in the right panel are for data-generating mechanism 2

**Fig. 14:**
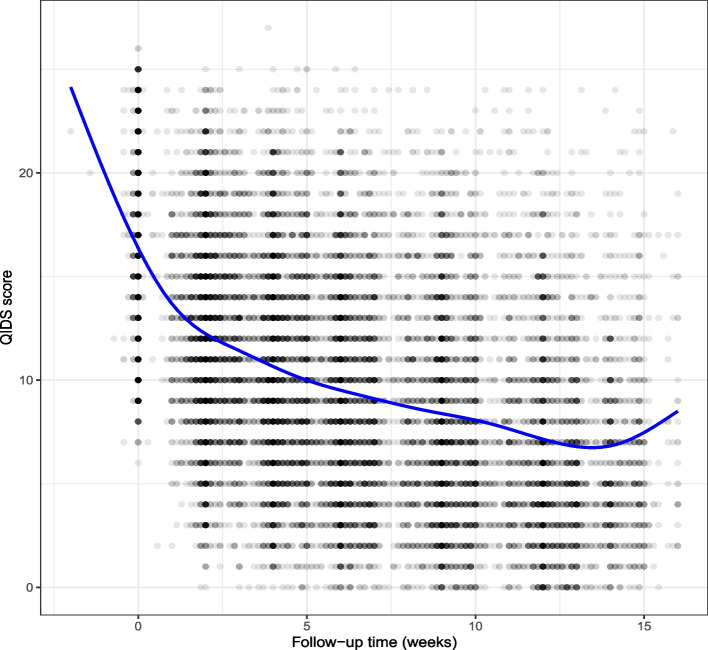
QIDS score trajectory over time fitted using smoothed conditional means

## Data Availability

Data used in the preparation of this manuscript were obtained from the National Institute of Mental Health (NIMH) Data Archive (NDA). NDA is a collaborative informatics system created by the National Institutes of Health to provide a national resource to support and accelerate research in mental health. Dataset identifier: 10.15154/1527974. This manuscript reflects the views of the authors and may not reflect the opinions or views of the NIH or of the Submitters submitting original data to NDA.

## 1 Theoretical results

### Bias in $\hat{\gamma }^{FU}$

In this section we consider the partial score function for $\gamma$ when people with no follow-up assessments are excluded i.e., we only include people whose number of follow-up assessments is larger than zero, expressed by $N_i(\tau )>0$ statistically.

We get a revised pseudo-score function of $\gamma$:

$$\begin{aligned} V^*(\gamma ) = \sum \limits _{i:N_i(\tau )>0} \int _0^\tau \left( \textbf{Z}_i(t) - \frac{\sum _{j:N_j(\tau )>0} Z_j(t){\textrm{exp}}(\gamma ^{\prime } Z_j(t))}{\sum _{j:N_j(\tau )>0} {\textrm{exp}}(\gamma ^{\prime } Z_j(t))}\right) dN_i(t) \end{aligned}$$

Define

$$\begin{aligned} n= & \text {number of subjects in dataset}\\ \bar{Z}_0(t;\gamma )= & \left( \sum \limits _{j} {\textrm{exp}}(\gamma ^{\prime } Z_j(t))\right) /n\\ \bar{Z}_1(t;\gamma )= & \left( \sum \limits _{j} Z_j(t){\textrm{exp}}(\gamma ^{\prime } Z_j(t))\right) /n\\ m= & \text {number of subjects with follow-up assessments in dataset}\\ \bar{Z}_0^*(t;\gamma )= & \left( \sum \limits _{j:N_j(\tau )>0} {\textrm{exp}}(\gamma ^{\prime } Z_j(t))\right) /m\\ \bar{Z}_1^*(t;\gamma )= & \left( \sum \limits _{j:N_j(\tau )>0} Z_j(t){\textrm{exp}}(\gamma ^{\prime } Z_j(t))\right) /m \end{aligned}$$

Then the estimating equation of $\gamma$ can be written as:

$$\begin{aligned} V(\gamma ) = \sum \limits _{i} \int _0^\tau \left( \textbf{Z}_i(t) - \frac{\bar{Z}_1(t;\gamma )}{\bar{Z}_0(t;\gamma )}\right) dN_i(t) \end{aligned}$$

and estimating equation of omitting subjects with no follow-up assessments is:

$$\begin{aligned} V^*(\gamma ) = \sum \limits _{i:N_i(\tau )>0} \int _0^\tau \left( \textbf{Z}_i(t) - \frac{\bar{Z}_1^*(t;\gamma )}{\bar{Z}_0^*(t;\gamma )}\right) dN_i(t) \end{aligned}$$

We know that

$$\begin{aligned} E\left( V^*(\varvec{\gamma }_0)-V(\varvec{\gamma }_0)\right) =E(V^*(\varvec{\gamma }_0))-E(V(\varvec{\gamma }_0))=E(V^*(\varvec{\gamma }_0)) \end{aligned}$$

Then we want to use the expectation of the difference of estimating equations between including all subjects and excluding subjects with no follow-up assessments to establish whether estimating equations when excluding subjects with no follow-up assessments also have zero mean.

A1 $$\begin{aligned} E\left( V^*(\gamma )-V(\gamma )\right)= & E\left( \sum \limits _{i} \int _0^\tau \left( \textbf{Z}_i(t) - \frac{\bar{Z}_1(t;\gamma )}{\bar{Z}_0(t;\gamma )}\right) dN_i(t)\right. \nonumber \\ & \left. -\sum \limits _{i:N_i(\tau )>0} \int _0^\tau \left( \textbf{Z}_i(t) - \frac{\bar{Z}_1^*(t;\gamma )}{\bar{Z}_0^*(t;\gamma )}\right) dN_i(t)\right) \nonumber \\= & E\left( \sum \limits _{i} \int _0^\tau \left( \textbf{Z}_i(t) - \frac{\bar{Z}_1(t;\gamma )}{\bar{Z}_0(t;\gamma )}\right) dN_i(t)\right. \nonumber \\ & \left. -\sum \limits _{i} \int _0^\tau \left( \textbf{Z}_i(t)- \frac{\bar{Z}_1^*(t;\gamma )}{\bar{Z}_0^*(t;\gamma )}\right) dN_i(t)\right) \nonumber \\= & E\left( \sum \limits _{i} \int _0^\tau \left( \frac{\bar{Z}_1^*(t;\gamma )}{\bar{Z}_0^*(t;\gamma )}- \frac{\bar{Z}_1(t;\gamma )}{\bar{Z}_0(t;\gamma )}\right) dN_i(t)\right) \nonumber \\= & \sum \limits _{i} \int _0^\tau E\left( \frac{\bar{Z}_1^*(t;\gamma )}{\bar{Z}_0^*(t;\gamma )}- \frac{\bar{Z}_1(t;\gamma )}{\bar{Z}_0(t;\gamma )}dN_i(t)\right) \end{aligned}$$

By the Strong Law of Large Numbers (SLLN),

$$\begin{aligned} \lim _{n\rightarrow \infty }\bar{Z}_0(t;\gamma )= & s_0(t;\gamma )=E({\textrm{exp}}(\gamma ^{\prime } Z_j(t))) \\ \lim _{n\rightarrow \infty }\bar{Z}_1(t;\gamma )= & s_1(t;\gamma )=E(Z_j(t){\textrm{exp}}(\gamma ^{\prime } Z_j(t)))\\ \lim _{m\rightarrow \infty }\bar{Z}_0^*(t;\gamma )= & s_0^*(t;\gamma )=E({\textrm{exp}}(\gamma ^{\prime } Z_j(t))\mid N_j(\tau )>0) \\ \lim _{m\rightarrow \infty }\bar{Z}_1^*(t;\gamma )= & s_1^*(t;\gamma )=E(Z_j(t){\textrm{exp}}(\gamma ^{\prime } Z_j(t))\mid N_j(\tau )>0) \end{aligned}$$

When *n* and *m* go to infinity, Eq. (A1) becomes,

$$\begin{aligned} & \sum \limits _{i} \int _0^\tau E\left( \frac{s_1^*(t;\gamma )}{s_0^*(t;\gamma )}- \frac{s_1(t;\gamma )}{s_0(t;\gamma )}dN_i(t)\right) \\= & \sum \limits _{i} \int _0^\tau E\left( \left( \frac{s_1^*(t;\gamma )}{s_0^*(t;\gamma )}- \frac{s_1(t;\gamma )}{s_0(t;\gamma )}\right) {\textrm{exp}}(\gamma ^\prime \textbf{Z}_i(t))\right) \lambda _0(t)dt \end{aligned}$$

Therefore, $E(V^*(\gamma ))\ne 0$ and $\hat{\gamma }$ is asymptotically biased when omitting subjects who has no follow-up assessments.

### Effect of parameters on bias in $\hat{\gamma }^{FU}$

Changing values of parameters could impact the magnitude of difference in right hand side of Eq. (5). We consider the case where auxiliary covariates are time-invariant in this section. Then $Z_j$ becomes a vector of constants.

When $\gamma$ is zero, $N_j(\tau )$ and $Z_j$ are independent. Therefore, $\frac{s_1^*(\gamma )}{s_0^*(\gamma )}- \frac{s_1(\gamma )}{s_0(\gamma )}=0$, $E(V^*(\gamma ))=0$ and $\hat{\gamma }$ is asymptotically unbiased in this case.

When $\gamma$ is not equal to zero, let $\hat{\gamma }$ be the estimator of $\varvec{\gamma }_0$ derived by solving $V^*(\gamma )=0$. Bias of $\hat{\gamma }$ can be expressed using a first-order Taylor Expansion of $V^*(\hat{\gamma })$ about $V^*(\varvec{\gamma }_0)$, where $V^*(\hat{\gamma })=0$.

$$\begin{aligned} V^*(\hat{\gamma })=V^*(\varvec{\gamma }_0)+\frac{\partial V(\varvec{\gamma }_0)}{\partial \varvec{\gamma }_0}(\hat{\gamma }-\varvec{\gamma }_0)+O(\hat{\gamma }-\varvec{\gamma }_0) \end{aligned}$$

Dividing by $\frac{1}{\sqrt{n}}$ on two sides, we have to first order

$$\begin{aligned} \frac{1}{\sqrt{n}}V^*(\hat{\gamma })=\frac{1}{\sqrt{n}}V^*(\varvec{\gamma }_0)+\frac{1}{n}\frac{\partial V(\varvec{\gamma }_0)}{\partial \varvec{\gamma }_0}\sqrt{n}(\hat{\gamma }-\varvec{\gamma }_0) \end{aligned}$$

Rearranging and taking expectations on two sides, we can get

$$\begin{aligned} E\left( \sqrt{n}(\hat{\gamma }-\varvec{\gamma }_0)\right) =E\left[ \left( \frac{1}{n}\frac{\partial V^*(\varvec{\gamma }_0)}{\partial \varvec{\gamma }_0}\right) ^{-1}\left( \frac{1}{\sqrt{n}}(V^*(\hat{\gamma })-V^*(\varvec{\gamma }_0))\right) \right] \end{aligned}$$

where $\frac{1}{n}\frac{\partial V^*(\varvec{\gamma }_0)}{\partial \varvec{\gamma }_0}$ converges almost surely to a constant by the SLLN. Therefore, the bias of $\hat{\gamma }$ can be written as

$$\begin{aligned} E(\hat{\gamma }-\varvec{\gamma }_0)= & \frac{1}{\sqrt{n}}\left( E\left( \frac{1}{n}\frac{\partial V^*(\varvec{\gamma }_0)}{\partial \varvec{\gamma }_0}\right) \right) ^{-1} E\left( \frac{1}{\sqrt{n}}(V^*(\hat{\gamma })-V^*(\varvec{\gamma }_0))\right) \\= & -\frac{1}{\sqrt{n}}\left( E\left( \frac{1}{n}\frac{\partial V^*(\varvec{\gamma }_0)}{\partial \varvec{\gamma }_0}\right) \right) ^{-1} E\left( \frac{1}{\sqrt{n}}V^*(\varvec{\gamma }_0)\right) \end{aligned}$$

Define

$$\begin{aligned} \bar{Z}_2^*(\gamma ) = \left( \sum _{j:N_j(\tau )>0} Z_j^\prime Z_j{\textrm{exp}}(\gamma ^{\prime } Z_j)\right) /m \end{aligned}$$

By the SLLN,

$$\begin{aligned} \lim _{m\rightarrow \infty }\bar{Z}_2^*(\gamma )=s_2^*(\gamma ) = E(Z_j^\prime Z_j{\textrm{exp}}(\gamma ^{\prime } Z_j)\mid N_j(\tau )>0) \end{aligned}$$

We assume *n* and *m* go to infinity. The expectation of $\frac{1}{n}\frac{\partial V^*(\varvec{\gamma }_0)}{\partial \varvec{\gamma }_0}$ can be written as

$$\begin{aligned} & E\left( \sum _i \frac{1}{n}\int _0^\tau \left( \frac{s_1^*(\varvec{\gamma }_0)}{s_0^*(\varvec{\gamma }_0)}\right) ^2-\frac{s_2^*(\varvec{\gamma }_0)}{s_0^*(\varvec{\gamma }_0)} dN_i(t)\right) \\= & \frac{1}{n}\sum _i \int _0^\tau E\left( \left( \left( \frac{s_1^*(\varvec{\gamma }_0)}{s_0^*(\varvec{\gamma }_0)}\right) ^2-\frac{s_2^*(\varvec{\gamma }_0)}{s_0^*(\varvec{\gamma }_0)}\right) {\textrm{exp}}(\textbf{Z}_i\gamma )\right) \lambda _0(t) dt \end{aligned}$$

If $\lambda _0$ is constant over time, the bias of $\hat{\gamma }$ can be written as

$$\begin{aligned} & -\left( \frac{1}{n}\sum _i E\left( \left( \left( \frac{s_1^*(\varvec{\gamma }_0)}{s_0^*(\varvec{\gamma }_0)}\right) ^2-\frac{s_2^*(\varvec{\gamma }_0)}{s_0^*(\varvec{\gamma }_0)}\right) {\textrm{exp}}(\textbf{Z}_i\gamma )\right) \lambda _0 \tau \right) ^{-1}\nonumber \\ & \left( \frac{1}{n}\sum _{i} E\left( \left( \frac{s_1^*(\varvec{\gamma }_0)}{s_0^*(\varvec{\gamma }_0)}- \frac{s_1(\varvec{\gamma }_0)}{s_0(\varvec{\gamma }_0)}\right) {\textrm{exp}}(\textbf{Z}_i\gamma )\right) \lambda _0\tau \right) \nonumber \\= & -\left( \left( \frac{s_1^*(\varvec{\gamma }_0)}{s_0^*(\varvec{\gamma }_0)}\right) ^2-\frac{s_2^*(\varvec{\gamma }_0)}{s_0^*(\varvec{\gamma }_0)}\right) ^{-1} \left( \frac{s_1^*(\varvec{\gamma }_0)}{s_0^*(\varvec{\gamma }_0)}- \frac{s_1(\varvec{\gamma }_0)}{s_0(\varvec{\gamma }_0)}\right) \end{aligned}$$

### Bias in $\hat{\beta }^{FU}$

In order to determine whether the estimated $\beta$ is biased when omitting subjects with no follow-up assessments, consider the IIW-GEE estimating equation evaluated at $\gamma =\hat{\gamma }$:

A2 $$\begin{aligned} U^*(\beta ;\hat{\gamma }) = \sum _{i:N_i(\tau )>0}\int _0^\tau \textbf{X}_i(t)^\prime (Y_i(t)-\textbf{X}_i(t)\beta ){\textrm{exp}}(-\textbf{Z}_i(t)\hat{\gamma })dN_i(t) \end{aligned}$$

Notice that for an arbitrary estimate $\tilde{\gamma }$ of $\gamma$ we have

$$\begin{aligned} & {\textrm{exp}}(-\textbf{Z}_i(t)\tilde{\gamma }) \\= & {\textrm{exp}}(-\textbf{Z}_i(t)\varvec{\gamma }_0) + {\textrm{exp}}(-\textbf{Z}_i(t)\varvec{\gamma }_0 - \textbf{Z}_i(t)\tilde{\gamma })({\textrm{exp}}(\textbf{Z}_i(t)\varvec{\gamma }_0) - {\textrm{exp}}(\textbf{Z}_i(t)\tilde{\gamma }))\\= & {\textrm{exp}}(-\textbf{Z}_i(t)\varvec{\gamma }_0) + {\textrm{exp}}(- \textbf{Z}_i(t)\varvec{\gamma }_0)( {\textrm{exp}}(\textbf{Z}_i(t)(\varvec{\gamma }_0-\tilde{\gamma }))-1) \end{aligned}$$

Now plug this into the Equation (A2) to get

A3 $$\begin{aligned} & \sum _{i:N_i(\tau )>0}\int _0^\tau \textbf{X}_i(t)^\prime (Y_i(t)-\textbf{X}_i(t)\beta )\left( {\textrm{exp}}(-\textbf{Z}_i(t)\varvec{\gamma }_0)\right. \nonumber \\ & \left. + {\textrm{exp}}(- \textbf{Z}_i(t)\varvec{\gamma }_0)( {\textrm{exp}}(\textbf{Z}_i(t)(\varvec{\gamma }_0-\tilde{\gamma }))-1)\right) dN_i(t) \end{aligned}$$

We examine the mean of this expression to check for bias in $\hat{\beta }$. Given

$$\begin{aligned} & E\left( \sum _{i:N_i(\tau )>0}\int _0^\tau \textbf{X}_i(t)^\prime (Y_i(t)-\textbf{X}_i(t)\beta ){\textrm{exp}}(-\textbf{Z}_i(t)\varvec{\gamma }_0) dN_i(t)\right) \\= & \sum _{i}\int _0^\tau E\left( \textbf{X}_i(t)^\prime (Y_i(t)-\textbf{X}_i(t)\beta ){\textrm{exp}}(-\textbf{Z}_i(t)\varvec{\gamma }_0) dN_i(t)\right) =0 \end{aligned}$$

and conditional independence of $Y_i(t)$ and $dN_i(t)$ given $\textbf{Z}_i(t)$, expectation of $U^*(\beta ;\tilde{\gamma })$ can be written as:

$$\begin{aligned} & E\left( \sum _i\int _0^\tau \textbf{X}_i(t)^\prime (Y_i(t)-\textbf{X}_i(t)\beta ){\textrm{exp}}(-\textbf{Z}_i(t)\tilde{\gamma })dN_i(t)\right) \\= & \sum _{i}\int _0^\tau E\left( \textbf{X}_i(t)^\prime (Y_i(t)-\textbf{X}_i(t)\beta )\right. \\ & \left( \textrm{exp}(- \textbf{Z}_{i}(t)\varvec{\gamma }_{0})(\textrm{exp}(\textbf{Z}_{i}(t)(\varvec{\gamma }_{0} - \tilde{\gamma })) - 1))\lambda _{0}(t)\textrm{exp}(\textbf{Z}_{i}(t)\varvec{\gamma }_{0})\right) dt\\= & \sum _{i}\int _{0}^{\tau } E\left( \textbf{X}_{i}(t)^{\prime } (Y_{i}(t)-\textbf{X}_{i}(t)\beta ) ({\textrm{exp}}(\textbf{Z}_{i}(t)(\varvec{\gamma }_{0} - \tilde{\gamma })) - 1)\right) \lambda _{0}(t)dt \end{aligned}$$

Using first order Taylor series to expand ${\textrm{exp}}(\textbf{Z}_i(t)(\varvec{\gamma }_0-\tilde{\gamma })$:

$$\begin{aligned} {\textrm{exp}}(\textbf{Z}_i(t)(\varvec{\gamma }_0-\tilde{\gamma }))= 1 + \textbf{Z}_i(t)(\varvec{\gamma }_0-\tilde{\gamma }) + O((\textbf{Z}_i(t)(\varvec{\gamma }_0-\tilde{\gamma }))^2) \end{aligned}$$

We aim to assess the impact of using $\tilde{\gamma }= \hat{\gamma }$:

$$\begin{aligned} {\textrm{exp}}(\textbf{Z}_i(t)(\varvec{\gamma }_0-\hat{\gamma })) = 1 + \textbf{Z}_i(t)(\varvec{\gamma }_0-\hat{\gamma }) + O((\textbf{Z}_i(t)(\varvec{\gamma }_0-\hat{\gamma }))^2) \end{aligned}$$

Then we can get to first order:

A4 $$\begin{aligned} E(U^*(\beta ;\hat{\gamma }))=\sum _i\int _0^\tau E\left( \textbf{X}_i(t)^\prime (Y_i(t)-\textbf{X}_i(t)\beta ) (-\textbf{Z}_i(t)(\hat{\gamma }-\varvec{\gamma }_0)) \right) \lambda _0(t)dt \end{aligned}$$

Since $\hat{\gamma }$ is asymptotically biased when omitting subjects with no follow-up assessments, $E(\hat{\gamma }-\varvec{\gamma }_0)\ne 0$. Therefore, $E(U^*(\beta ;\hat{\gamma }))\ne 0$ and $\hat{\beta }$ is asymptotically biased when omitting subjects with no follow-up assessments.

### Effect of parameters on bias in $\hat{\beta }^{FU}$

Let $\hat{\beta }$ be the estimator of $\varvec{\beta }_0$ derived by solving $U^*(\beta ;\hat{\gamma })=0$. Bias of $\hat{\beta }$ can be expressed using the first-order Taylor Expansion of $U^*(\hat{\beta };\hat{\gamma })$ about $U^*(\varvec{\beta }_0;\hat{\gamma })$, where $U^*(\hat{\beta };\hat{\gamma })=0$.

$$\begin{aligned} U^*(\hat{\beta };\hat{\gamma }) = U^*(\varvec{\beta }_0;\hat{\gamma }) + \frac{\partial U^*(\varvec{\beta }_0;\hat{\gamma })}{\partial \varvec{\beta }_0}(\hat{\beta }-\varvec{\beta }_0) + O(\hat{\beta }-\varvec{\beta }_0) \end{aligned}$$

Dividing by $\frac{1}{\sqrt{n}}$ on two sides,

$$\begin{aligned} \frac{1}{\sqrt{n}}U^*(\hat{\beta };\hat{\gamma })=\frac{1}{\sqrt{n}}U^*(\varvec{\beta }_0;\hat{\gamma })+\frac{1}{n}\frac{\partial U^*(\varvec{\beta }_0;\hat{\gamma })}{\partial \varvec{\beta }_0}\sqrt{n}(\hat{\beta }-\varvec{\beta }_0) \end{aligned}$$

Rearranging and taking expectations on two sides, we can get

$$\begin{aligned} E\left( \sqrt{n}(\hat{\beta }-\varvec{\beta }_0)\right) =E\left[ \left( \frac{1}{n}\frac{\partial U^*(\varvec{\beta }_0;\hat{\gamma })}{\partial \varvec{\beta }_0}\right) ^{-1}\left( \frac{1}{\sqrt{n}}(U^*(\hat{\beta };\hat{\gamma })-U^*(\varvec{\beta }_0;\hat{\gamma }))\right) \right] \end{aligned}$$

where $\frac{1}{n}\frac{\partial U^*(\varvec{\beta }_0;\hat{\gamma })}{\partial \varvec{\beta }_0}$ converges almost surely to a constant by the SLLN. Therefore, the bias of $\hat{\beta }$ can be written as

$$\begin{aligned} E(\hat{\beta }-\varvec{\beta }_0)= & \frac{1}{\sqrt{n}}\left( E\left( \frac{1}{n}\frac{\partial U^*(\varvec{\beta }_0;\hat{\gamma })}{\partial \varvec{\beta }_0}\right) \right) ^{-1}E\left( \frac{1}{\sqrt{n}}(U^*(\hat{\beta };\hat{\gamma })-U^*(\varvec{\beta }_0;\hat{\gamma }))\right) \\= & -\frac{1}{\sqrt{n}}\left( E\left( \frac{1}{n}\frac{\partial U^*(\varvec{\beta }_0;\hat{\gamma })}{\partial \varvec{\beta }_0}\right) \right) ^{-1}E\left( \frac{1}{\sqrt{n}}U^*(\varvec{\beta }_0;\hat{\gamma })\right) \end{aligned}$$

where

$$\begin{aligned} & E\left( \frac{1}{n}\frac{\partial U^*(\varvec{\beta }_0;\hat{\gamma })}{\partial \varvec{\beta }_0}\right) =E\left( -\frac{1}{n}\sum _i \int _0^\tau \textbf{X}_i(t)^\prime \textbf{X}_i(t) {\textrm{exp}}(\textbf{Z}_i(t)\hat{\gamma })dN_i(t) \right) \\= & -\frac{1}{n}\sum _i \int _0^\tau E\left( \textbf{X}_i(t)^\prime \textbf{X}_i(t) {\textrm{exp}}(\textbf{Z}_i(t)\hat{\gamma }){\textrm{exp}}(\textbf{Z}_i(t)\varvec{\gamma }_0)\right) \lambda _0(t) dt \end{aligned}$$

If $\textbf{Z}_i$ and $\lambda _0$ are time-independent, the bias of $\hat{\beta }$ can be written as

A5 $$\begin{aligned} & \left( \frac{1}{n}\sum _i\int _0^\tau E\left( \textbf{X}_i(t)^\prime \textbf{X}_i(t) {\textrm{exp}}(\textbf{Z}_i\hat{\gamma }){\textrm{exp}}(\textbf{Z}_i\varvec{\gamma }_0)\right) dt\lambda _0 \right) ^{-1} \nonumber \\ & \left( \frac{1}{n}\sum _i \int _0^\tau E\left( \textbf{X}_i(t)^\prime (Y_i(t) -\textbf{X}_i(t)\varvec{\beta }_0)(-\textbf{Z}_i(\hat{\gamma }-\varvec{\gamma }_0)) \right) dt\lambda _0 \right) \nonumber \\= & \left( \sum _i \int _0^\tau E\left( \textbf{X}_i(t)^\prime \textbf{X}_i(t) {\textrm{exp}}(\textbf{Z}_i\hat{\gamma }){\textrm{exp}}(\textbf{Z}_i\varvec{\gamma }_0)\right) dt\right) ^{-1} \nonumber \\ & \left( \sum _i \int _0^\tau E\left( \textbf{X}_i(t)^\prime (Y_i(t)-\textbf{X}_i(t)\varvec{\beta }_0) (-\textbf{Z}_i(\hat{\gamma }-\varvec{\gamma }_0))\right) dt\right) \end{aligned}$$

## 2 Supplementary simulation results

### Effect of large *n*

Simulation results support the hypothesis that bias is not a function of sample size, provided that sample size is sufficient that paramters can be estimated with minimal bias on including everyone. In Fig. 11, the bias lines of $\hat{\text {AUC}}$ and $\hat{\gamma }$ are almost horizontal which means increasing *n* does not lead to a decrease of bias of $\hat{\text {AUC}}$ and $\hat{\gamma }$ for either data-generating mechanism. In this case, *n* is large enough ($\ge$100) to estimate parameters with minimal bias on including all subjects. Bias of $\hat{\text {AUC}}$ and $\hat{\gamma }$ are stable and close to zero when including everyone.

### Tabulated simulation results

The following tables summarize the simulation results in Simulation section.

**Table 4:** Summary of Bias, Estimated Standard Errors (ESE: standard deviation of the 5000 estimates) and Average Standard Errors (ASE: mean of the 5000 estimates of standard errors) in AUC and $\gamma$ for data-generating mechanism 1

	Including all people						Omitting people with no follow-up assessments						Mean	
	AUC			$\gamma$			AUC			$\gamma$			number	Probability
Parameter	Bias	ESE	ASE	Bias	ESE	ASE	Bias	ESE	ASE	Bias	ESE	ASE	of visits	$N_i(\tau )=0$
$$\tau$$														
1	-0.00	0.10	0.10	0.00	0.10	0.10	-0.14	0.11	0.12	0.37	0.10	0.11	1.28	0.25
1.5	-0.00	0.13	0.14	0.00	0.07	0.07	-0.12	0.14	0.14	0.21	0.07	0.08	1.80	0.12
2	-0.00	0.16	0.17	0.00	0.06	0.06	-0.09	0.16	0.18	0.11	0.06	0.06	2.29	0.06
2.5	-0.00	0.20	0.21	0.00	0.05	0.05	-0.07	0.20	0.22	0.06	0.05	0.06	2.75	0.03
3	-0.00	0.25	0.26	0.00	0.05	0.05	-0.05	0.25	0.27	0.03	0.05	0.05	3.21	0.02
$$\lambda _0$$														
0.81	0.00	0.15	0.16	0.00	0.05	0.05	-0.02	0.15	0.16	0.02	0.05	0.05	3.52	0.01
0.55	0.00	0.16	0.17	0.00	0.06	0.06	-0.07	0.16	0.17	0.09	0.06	0.06	2.49	0.05
0.36	-0.00	0.18	0.18	0.00	0.07	0.07	-0.18	0.19	0.20	0.23	0.07	0.08	1.72	0.13
0.24	-0.00	0.20	0.21	0.00	0.09	0.09	-0.31	0.22	0.24	0.43	0.09	0.10	1.20	0.26
0.16	0.00	0.22	0.23	0.00	0.12	0.11	-0.44	0.28	0.30	0.67	0.12	0.12	0.83	0.40
$$\gamma _0$$														
0.0	-0.00	0.21	0.21	0.00	0.08	0.09	-0.48	0.23	0.24	0.62	0.10	0.10	1.00	0.37
0.2	-0.00	0.19	0.19	0.00	0.07	0.07	-0.30	0.20	0.21	0.38	0.08	0.08	1.42	0.22
0.4	-0.00	0.17	0.18	0.00	0.07	0.07	-0.15	0.18	0.18	0.19	0.07	0.07	1.96	0.10
0.6	-0.00	0.16	0.17	0.00	0.06	0.06	-0.05	0.16	0.17	0.06	0.06	0.06	2.65	0.04
0.8	-0.00	0.15	0.16	0.00	0.06	0.06	-0.01	0.15	0.16	0.01	0.06	0.06	3.52	0.01
*n*														
10	0.04	1.18	0.97	0.09	0.54	0.51	-0.01	1.20	0.98	0.21	0.55	0.52	2.29	0.06
20	0.02	0.83	0.76	0.04	0.34	0.33	-0.05	0.85	0.77	0.15	0.34	0.34	2.29	0.06
30	0.00	0.67	0.65	0.02	0.27	0.26	-0.07	0.68	0.66	0.14	0.27	0.27	2.29	0.06
40	0.00	0.59	0.58	0.02	0.23	0.23	-0.08	0.60	0.59	0.13	0.23	0.23	2.29	0.06
50	-0.00	0.53	0.52	0.01	0.20	0.20	-0.09	0.53	0.53	0.13	0.20	0.21	2.28	0.06
100	-0.00	0.37	0.38	0.01	0.14	0.14	-0.09	0.37	0.39	0.12	0.14	0.15	2.28	0.06
200	-0.00	0.26	0.27	0.00	0.10	0.10	-0.09	0.26	0.28	0.12	0.10	0.10	2.28	0.06
300	0.00	0.21	0.22	0.00	0.08	0.08	-0.09	0.21	0.23	0.11	0.08	0.08	2.29	0.06
400	-0.00	0.18	0.19	0.00	0.07	0.07	-0.09	0.18	0.20	0.11	0.07	0.07	2.29	0.06
500	-0.00	0.16	0.17	0.00	0.06	0.06	-0.09	0.16	0.18	0.11	0.06	0.06	2.29	0.06

**Table 5:** Summary of Bias, Estimated Standard Errors (ESE: standard deviation of the 5000 estimates) and Average Standard Errors (ASE: mean of the 5000 estimates of standard errors) in AUC and $\gamma$ for data-generating mechanism 2

	Including all people						Omitting people with no follow-up assessments						Mean	
	AUC			$\gamma$			AUC			$\gamma$			number	Probability
Parameter	Bias	ESE	ASE	Bias	ESE	ASE	Bias	ESE	ASE	Bias	ESE	ASE	of visits	$N_i(\tau )=0$
1	0.00	0.10	0.11	0.00	0.09	0.09	0.18	0.11	0.11	-0.27	0.06	0.09	1.00	0.38
1.5	0.00	0.13	0.14	0.00	0.07	0.07	0.18	0.14	0.14	-0.21	0.06	0.07	1.50	0.24
2	-0.00	0.16	0.18	0.00	0.06	0.06	0.17	0.17	0.17	-0.16	0.05	0.06	1.99	0.15
2.5	-0.00	0.20	0.22	0.00	0.05	0.06	0.17	0.20	0.21	-0.12	0.05	0.06	2.48	0.10
3	0.00	0.25	0.27	-0.00	0.05	0.05	0.16	0.25	0.26	-0.09	0.05	0.05	2.97	0.07
$$\lambda _0$$														
0.81	0.00	0.15	0.16	-0.00	0.05	0.05	0.10	0.15	0.16	-0.08	0.05	0.05	3.21	0.05
0.55	-0.00	0.16	0.17	0.00	0.06	0.06	0.16	0.17	0.17	-0.14	0.05	0.06	2.19	0.13
0.36	0.00	0.18	0.19	0.00	0.08	0.07	0.22	0.18	0.19	-0.21	0.06	0.08	1.44	0.25
0.24	0.01	0.21	0.22	-0.00	0.09	0.09	0.25	0.22	0.21	-0.26	0.07	0.09	0.97	0.39
0.16	0.01	0.25	0.26	0.00	0.11	0.11	0.27	0.26	0.25	-0.29	0.08	0.11	0.65	0.53
$$\gamma _0$$														
0	0.00	0.20	0.21	-0.00	0.08	0.08	0.01	0.21	0.21	-0.01	0.06	0.08	1.00	0.37
0.2	-0.00	0.19	0.19	0.00	0.07	0.07	0.09	0.20	0.19	-0.09	0.06	0.07	1.31	0.27
0.4	-0.00	0.17	0.18	-0.00	0.07	0.07	0.15	0.18	0.18	-0.14	0.06	0.07	1.73	0.19
0.6	-0.00	0.16	0.17	0.00	0.06	0.06	0.18	0.16	0.17	-0.16	0.05	0.06	2.29	0.12
0.8	-0.00	0.16	0.17	0.00	0.05	0.05	0.18	0.15	0.16	-0.15	0.05	0.06	3.04	0.08
*n*														
10	0.05	1.21	1.00	0.05	0.56	0.51	0.17	1.21	1.00	-0.12	0.51	0.53	1.99	0.15
20	0.02	0.84	0.79	0.02	0.35	0.34	0.17	0.84	0.79	-0.14	0.30	0.34	1.99	0.15
30	0.01	0.69	0.67	0.01	0.28	0.27	0.17	0.69	0.66	-0.15	0.24	0.27	1.99	0.15
40	0.01	0.59	0.59	0.01	0.23	0.23	0.17	0.60	0.59	-0.15	0.20	0.23	1.99	0.15
50	0.01	0.53	0.54	0.01	0.20	0.20	0.17	0.53	0.53	-0.15	0.18	0.21	1.99	0.15
100	0.00	0.37	0.39	0.00	0.14	0.14	0.17	0.38	0.38	-0.16	0.12	0.14	1.99	0.15
200	-0.00	0.26	0.28	0.00	0.10	0.10	0.17	0.27	0.27	-0.16	0.09	0.10	1.99	0.15
300	-0.00	0.22	0.23	0.00	0.08	0.08	0.17	0.22	0.22	-0.16	0.07	0.08	1.99	0.15
400	-0.00	0.19	0.20	0.00	0.07	0.07	0.17	0.19	0.19	-0.16	0.06	0.07	1.99	0.15
500	-0.00	0.16	0.18	0.00	0.06	0.06	0.17	0.17	0.17	-0.16	0.05	0.06	1.99	0.15

## 3 Supplementary STAR*D results

**Table 6:** Demographic characteristics of the 4041 individuals in the STAR*D cohort at baseline. Hope (enjoyment) was captured through responses (agree/neutral/disagree) to the statement “If I can get the help I need from a doctor, I believe that I will be much better able to enjoy things”; Hope (decision-making) was captured through responses (agree/neutral/disagree) to the statement “If I can get the help I need from a doctor, I believe that I will be better able to make important decisions”

Characteristic	Number (%)	Missing
**Male**	1509 (37%)	2 (0.0%)
**Age in decades**		
Median [Min, Max]	4.05 [1.81, 7.57]	4 (0.0%)
**Married**	1332 (33%)	4 (0.0%)
**Currently a student**	593 (15%)	4 (0.0%)
**Current employment status**		
Employed	2291 (57%)	
Unemployed	1478 (37%)	39 (0.1%)
Retired	233 (6%)	
**Total number of persons in household**		
>2	1986 (49%)	10 (0.0%)
**Number of decades in formal education**		
Median [Min, Max]	1.30 [0, 2.70]	14 (0.0%)
**On medical or psychiatric leave**	312 (8%)	11 (0.0%)
**Hope: enjoyment**		
Agree	3690 (92%)	
Neutral	298 (7%)	18 (0.0%)
Disagree	35 (1%)	
**Hope: decision-making**		
Agree	3650 (91%)	
Neutral	327 (8%)	18 (0.0%)
Disagree	46 (1%)	
**Impact of family & friends**		
Helpful	2345 (58%)	
Neutral	776 (19%)	20 (0.0%)
Difficult	900 (22%)

**Table 7:** Estimated regression coefficients (standard errors) for the fitted mean outcome trajectory of QIDS scores including all subjectsm and excluding subjects with no follow-up assessments. $\varvec{\beta }_0$ and $\beta _1$ are regression coefficients for intercept and log(1+time) separately. No other covariates were included in this model

	Including everyone	Excluding subjects with no follow-up assessments
Intercept	16.35 (0.061)	16.33 (0.064)
$\log (1+\text {time})$	-3.09 (0.042)	-3.23 (0.040)

**Table 8:** Estimated main effects from the model of change from baseline in QIDS scores; interaction terms from this same model are shown in Table 2

Predictor	Level or	Estimate (SE)	
	Units	Everyone	$\ge 1$ follow-up assessment
Male		0.31 (0.15)	0.29 (0.15)
Age	Decades	0.34 (0.07)	0.34 (0.07)
Residence	House	-0.47 (0.18)	-0.45 (0.17)
reference: Apartment/Condo	Other	0.49 (0.28)	0.51 (0.28)
Marital Status	Married	0.09 (0.17)	0.08 (0.17)
$> 2$ people in household		0.38 (0.17)	0.34 (0.16)
Student		0.01 (0.21)	0.95 (0.21)
Employment	Retired	-0.02 (0.34)	-0.01 (0.33)
reference: Employed	Unemployed	0.46 (0.17)	0.47 (0.16)
Volunteer work	Yes	-0.02 (0.21)	-0.03 (0.21)
Medical/psychiatric leave		0.80 (0.30)	0.72 (0.30)
Has private insurance	Yes	-0.18 (0.13)	-0.16 (0.13)
Hope: Decision-making	Disagree	0.63 (0.76)	0.65 (0.75)
reference: Agree	Neutral	0.36 (0.29)	0.35 (0.29)
Hope: Enjoyment	Disagree	-1.07 (0.84)	-0.95 (0.81)
reference: Agree	Neutral	0.87 (0.31)	0.88 (0.31)
Impact of family & friends	Helpful	-0.44 (0.18)	-0.42 (0.18)
reference: Difficult	Neutral	-0.13 (0.23)	-0.11 (0.22)
Education	Decades	-0.13 (0.25)	-0.14 (0.24)

## Code availability

Code is provided in the Supplementary Materials.
